# Design of a Lightweight, Cost Effective Thimble-Like Sensor for Haptic Applications Based on Contact Force Sensors

**DOI:** 10.3390/s111211495

**Published:** 2011-12-06

**Authors:** Manuel Ferre, Ignacio Galiana, Rafael Aracil

**Affiliations:** Centre for Automation and Robotics CAR UPM-CSIC, Universidad Politécnica de Madrid, ETSII-Automática., C/. José Gutiérrez Abascal, 2, 28006 Madrid, Spain; E-Mails: ignacio.galiana@upm.es (I.G.); rafael.aracil@upm.es (R.A.)

**Keywords:** thimble, end-effector, normal and tangential forces, manipulation forces, force estimation, contact force sensors, virtual object manipulation

## Abstract

This paper describes the design and calibration of a thimble that measures the forces applied by a user during manipulation of virtual and real objects. Haptic devices benefit from force measurement capabilities at their end-point. However, the heavy weight and cost of force sensors prevent their widespread incorporation in these applications. The design of a lightweight, user-adaptable, and cost-effective thimble with four contact force sensors is described in this paper. The sensors are calibrated before being placed in the thimble to provide normal and tangential forces. Normal forces are exerted directly by the fingertip and thus can be properly measured. Tangential forces are estimated by sensors strategically placed in the thimble sides. Two applications are provided in order to facilitate an evaluation of sensorized thimble performance. These applications focus on: (i) force signal edge detection, which determines task segmentation of virtual object manipulation, and (ii) the development of complex object manipulation models, wherein the mechanical features of a real object are obtained and these features are then reproduced for training by means of virtual object manipulation.

## 1. Introduction

The capacity to measure forces exerted by a person during manipulation is highly valuable in relation to the performance of real manipulations [1] or virtual object manipulation [2]. The aim of the project described in this article is to show the capabilities of contact force sensors in estimating forces exerted by a person in both real and virtual manipulation tasks.

Force/torque transducers or load cells are usually used to obtain these types of measurements [3]. These devices are very precise; however, they have inconveniences, mainly related to their weight, volume, and high cost. The project developed in this paper focuses on using contact force sensors to estimate manipulation forces. Specifically, the contact force sensors referred to in this paper are FlexiForce model A201 by Tekscan Inc. This type of sensor acts as a force-sensing resistor, such that when the force sensor is unloaded, its resistance is very high, and when force is applied to the sensor, this resistance decreases. Several types of material show this kind of behavior, from metals to semiconductors [4]. The principle drawbacks of these sensors relate to low repeatability and hysteresis. Nevertheless, contact force sensors and piezoresistive sensors provide an effective solution for varying kinds of applications. Examples include: the detection of collisions in human-robot interaction [5], the measurement of manipulation forces [6–8], biomedical applications [9,10], and minimally invasive surgery [11] due to the sensors’ highly reduced size, weight, and cost. This paper proposes the design of an end-effector for haptic devices that incorporates contact force sensors in order to estimate manipulation forces when interacting with virtual or real environments.

Haptic interfaces are force feedback devices that enable bidirectional human-system interactions and provide the operator with force information from this interaction while simultaneously capturing the operator’s motion or force input [12]. These devices can be used to interact with virtual (virtual telepresence) or real (telepresence) environments. The principle applications of these systems concern teleoperation in remote environments such as tele-surgery or tele-manipulation, and virtual applications for advanced manual training techniques such as medical procedures or rehabilitation exercises.

It is widely accepted that haptic devices benefit from force measurement capabilities in terms of the reduction of device dynamics or an increase in the fidelity of the forces exerted to the user [13]. However, the use of load cells in haptic devices is limited as a result of inertia and cost considerations. Katsura *et al.* [14] note that the limited bandwidth and high cost of force sensors hinder their widespread acceptance in such applications. Previous studies use expensive, high precision, and high weight force/torque sensors attached to haptic devices to provide more reliable forces in complex tactile simulation applications [2] or for measuring and recording the material properties of soft objects [15]. Moreover, in the case of the aforementioned complex tactile simulation applications, the authors [2] recognize the importance of force sensing in haptic devices and point out that the use of force sensors significantly increases the price of their applications. Nonetheless, previous projects using haptic interfaces do not take into consideration the use of cost-effective thimble-like sensors as contact force sensors for estimating normal and tangential forces. Our work aims to develop a lightweight end-effector that provides force measurement capabilities to commercially available haptic devices.

This article is organized as follows: Section 2 describes the principal characteristics and requirements that a sensorized thimble must comply with in virtual manipulation applications that measure the force exerted by a user. These requirements are comparable to those used for the manipulation of real objects. Section 3 describes the mechanical design of the thimble and the location of the contact force sensors. Section 4 focuses on the calibration of the contact force sensors by means of a load cell and the calibration of the thimble with the four contact force sensors. The proper calibration of the sensors does not guarantee in and of itself the correct performance of the thimble as a whole, since finger deformations occurring as a result of an increase in the grip force need to be taken into account. Section 5 provides various examples of applications in which the sensorized thimble has been used with positive results. Finally, in Section 6 some conclusions about this project are provided.

## 2. Principle Requirements for a Thimble-Like End-Effector for Haptic Devices

In some cases, the thimble-like device has opposing compliance requirements for manipulation applications. Therefore, it is necessary to establish which objectives are most important and take into account that other objectives may be compromised. In haptic applications, the thimble represents the end-effector of the device. This means that the size and weight of the thimble must be as light as possible. When manipulating real objects, similar conditions exist given that, as the thimble increases in size the distortions in the forces applied become greater. Ideally, the thimble should not interfere with the user more than that of a medical glove. In sum, the principle requirements for a thimble-like device are as follows:

1. It must be adjustable to different-sized fingers,
2. The thimble must be as light as possible,
3. The user must feel comfortable using the device, and
4. The force securing the unit to the user’s finger must not affect the user’s perception.

The first requirement concerns whether to choose a thimble that can be adjusted to the user or to have a set of thimbles of different sizes thereby allowing each user to wear a more adequate thimble. The availability of different-sized thimbles initially appears advantageous. However, this is only advisable if the thimble is mainly used by the same person. For applications having several users who require different thimble sizes, the constant changing of thimbles is inconvenient and results in the deterioration of mechanical and electrical connections. The resulting loose connections and malfunctions in the electrical contacts lead to poor performance of the device. Thus, it is preferable to use a thimble that can be adjusted to the user’s finger. More components must be added in order to achieve such adjustments and is a drawback to the abovementioned second requirement. Nonetheless, the adaptability of the thimble to different users has priority.

The second previously mentioned requirement refers to the design of a thimble that is as light as possible. As we mentioned earlier, the thimble is located at the end-point of the device; therefore a minor additional weight could significantly increase the inertia (proportional to the square of the distance). In haptic interfaces, high inertia can lead to a dynamic distortion of the user’s perception [16,17]. This is the main reason why contact force sensors were considered in this design as opposed to traditional load cells. A load cell consists of a gauge inside a metal case, which results in a heavy and bulky design.

The third and fourth requirements relate to ergonomic considerations in order to achieve the least possible deterioration in the user’s perception. The force to which the thimble is adjusted in relation to the user’s finger is a critical factor in the performance of the thimble. This force must be minimal in order to avoid causing discomfort to or distortion in the user’s perception. However, the attachment must be sufficiently firm in order to ensure that the thimble is not dislodged from the finger during manipulation. Therefore, depending on the task performed and the force capabilities of the haptic device, the tightening mechanism between the thimble and finger may be adjusted.

## 3. Design of a Thimble-Like Device with Force Measurement Capabilities

### 3.1. Sensorized Thimble Configuration

**Figure 1.**
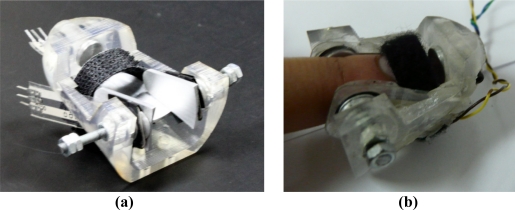
**(a)** Thimble: two screws allow the thimble to be adapted to different finger sizes. Four mechanically isolated contact force sensors are used for measuring the contact forces, **(b)** User wearing the thimble; the finger is secured by means of the screws and Velcro.

The thimble is designed to fit different-sized fingers by means of a screw system that adapts to the sides of the finger. Velcro is also used to hold the finger to the thimble. The thimble has a cone-shaped design, narrow at the top and a little thicker at the bottom at the point at which the distal phalanx joins the beginning of the middle phalanx. This geometry is similar to the human finger, thereby allowing natural human-object interaction during manipulation. The thimble design is shown in Figure 1.

The thimble was made out of an epoxy resin in order to reduce its weight. A technique known as stereolithography rapid prototyping (or stereolithography) was used in the manufacturing process [18]. Consequently, the thimble weighs 76 grams, which makes it ideal for virtual and real object manipulation. That is, lower weight implies lower inertia and, therefore, less interference with the user’s weight perception.

**Figure 2.**
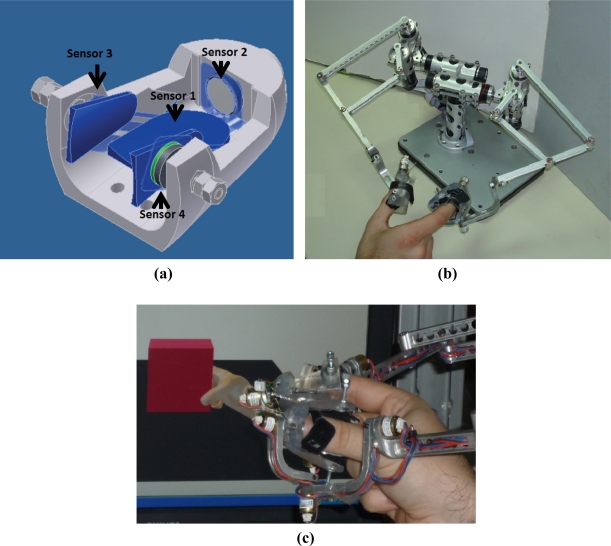
**(a)** Four piezoresistive sensors are used to estimate manipulation forces. Sensor 1 provides normal forces directly applied by the fingertip, and sensors 2, 3, and 4 estimate the tangential forces. **(b)** The user inserts the thumb and index finger in the corresponding thimbles and the thimbles are firmly linked to the haptic device. **(c)** The user grasps a virtual box. Forces applied by the user are captured by the sensorized thimbles.

The thimble includes four Flexiforce contact force sensors manufactured by Tekscan Inc. [19]. These sensors are used for estimating normal and tangential forces at the fingertip. Contact force sensors only provide the normal component force applied to its active area. Sensors are located in the thimble, thereby enabling the measurement of normal and tangential manipulation. Normal manipulation force is provided by the sensor located in the fingertip, as shown by the “Sensor 1” label in Figure 2. Three additional sensors are placed on the sides of the phalanx in order to estimate the tangential manipulation force. These sensors are labeled as “Sensor 2”, “Sensor 3”, and “Sensor 4” in Figure 2. This thimble is designed to fit a particular haptic device. Figure 2(b) shows how the thimble is attached to a two-finger haptic device called the MasterFinger-2 [20] by means of a screw system.

### 3.2. Sensor Interaction Due to Finger Deformation

In addition to the manipulation forces, the force used to secure the finger to the thimble affects sensors 3 and 4. That is, in addition to this clamping force, a small deformation in the finger appears when an object is grasped. This deformation significantly increases the pressure applied to the side sensors. Due to the symmetrical geometry of the thimble, finger deformations create the same force on either side sensor. These effects must be taken into account when estimating the real tangential force.

The finger deformation effect appears when force is applied by the fingertip. For instance, when pressing a surface in a normal direction, the sensor located at the bottom of the thimble (sensor 1) should be the only sensor to detect such forces. Nonetheless, forces are also detected by sensors 2 and 3 due to finger deformations.

**Figure 3.**
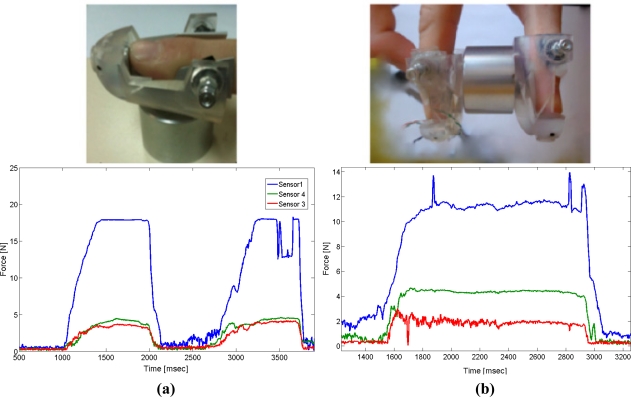
Data provided by sensors 1, 2, and 3. **(a)** Forces when pressing an object; **(b)** Forces when grasping a cylinder.

The effect is illustrated in Figure 3(a): a cylinder weighing 4.5 N was used to show forces caused by finger deformations within the thimble. The user applies a normal force to a rigid surface. Consequently, sensors 2 and 3 measure similar forces resulting from the symmetric deformation of the finger. In Figure 3(b), the cylinder is grasped horizontally using two thimbles. The sensor located under the fingertip measures the normal force that is applied to hold the cylinder, whereas the tangential force is estimated by the sensor located at the upper sides of the finger and equal half of the total weight of the cylinder, since two thimbles are used. The data provided by the sensors is shown in Figure 3(b). The highest force value is the one measured by sensor 1 (corresponding to the normal force exerted by the user). The subtraction of the two measurements from the lateral sensors represents the real tangential force. The total weight obtained is 4.3 − 2.0 = 2.3 N, which is a close approximation to half of the cylinder’s weight. Thus, the measurement provided by the contact force sensor with this configuration represents a close approximation to the expected value.

## 4. Calibration of Sensor and Thimble

### 4.1. Sensor Assembly and Its Calibration

**Figure 4.**
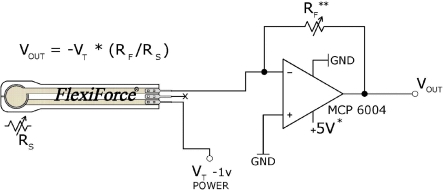
Electronic circuit for processing the contact force sensor signal.

A A201-25 Flexiforce sensor from Tekscan [19] with a range of 0 to 110 N was selected. The thickness of the sensor is approximately 0.2 mm and is very lightweight. According to the manufacturer specifications, the repeatability is ±2.5%. The active area of this sensor consists of an ultra-thin and flexible printed circuit located in a circle with a 9.53 mm diameter. Its behavior is similar to a variable resistor. When the sensor is unloaded, its resistance is very high (greater than 5 MΩ). This resistance decreases when force is applied to the active sensor area. The electronic circuit recommended by the manufacturer is shown in Figure 4.

**Figure 5.**
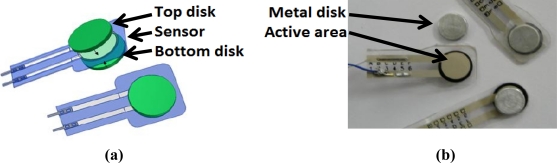
**(a)** Contact force sensor is placed between two metal disks in order to mechanically isolate the sensor, **(b)** various views of the sensors and disks.

The applied force must be homogeneously exerted over the active sensing area in order to guarantee proper sensor repeatability. For this reason, a “sandwich configuration” mechanical assembly was used to properly transmit forces to the sensor’s active area. This mounting consists in placing the sensor in the middle of two cylindrical metal sheets that are located over the active sensor area, as shown in Figure 5(a). The cylindrical metal sheets must have a very flat surface in order to obtain proper repeatability. This “sandwich configuration” guarantees mechanical isolation between the finger and the thimble, since the user force is thoroughly transmitted to the sensor. This configuration has been successfully tested and used in many experiments.

The assembly previously described (disk+sensor+disk) was calibrated by means of a high accuracy six-axis force/torque sensor manufactured by ATI Industrial Automation, model Nano17 [21]. This sensor provides six-dimensional force components (forces/torques). It consists of a monolithic transducer with a silicon strain gauge. A set of different weights was used to perform the calibration of the “sandwich configuration” assembly. Signals provided by both sensors (ATI-Nano 17 and Flexiforce-A201-25 in ‘sandwich configuration’) were processed for the different weight sets. The calibration was carried out using the least square polynomial interpolation. A monotone increasing polynomial was obtained that guarantees only one force value for each voltage provided by the contact force sensor. This polynomial interpolation improves manufacturer performance. The polynomial interpolation function obtained is as follows:

(1) $$f\left(v\right)=0.0557v^{7}-0.9616v^{6}+6.5957v^{5}-22.9525v^{4}+42.9525v^{3}-41.9706v^{2}+22.0111v+0.0648$$

where “v” is the voltage provided by the contact force sensor and “f” is the estimated value of the exerted force.

**Figure 6.**
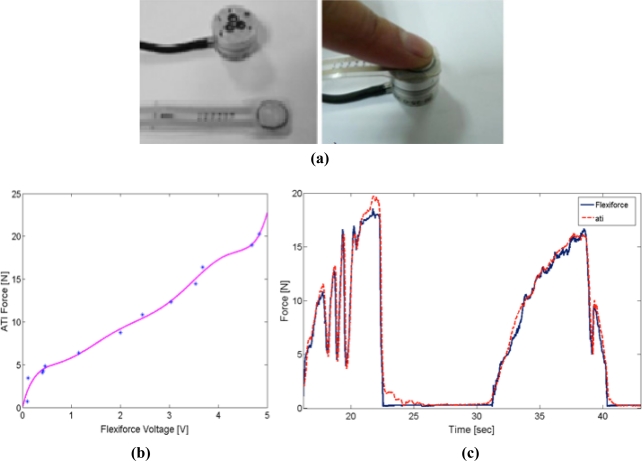
Contact force sensor assembly calibration. **(a)** Set-up for contact force sensor calibration using a Nano 17 ATI sensor, **(b)** Least square polynomial interpolation of the data, **(c)** Comparison between Flexiforce and ATI data.

As shown in Figure 6(c), the data provided by the contact force sensor and the Nano17 sensor is very similar and has a range of 0 to 20 N. This force range is usually sufficient for common manipulation tasks.

### 4.2. Thimble Calibration

**Figure 7.**
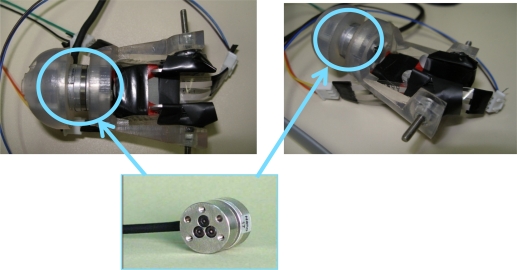
New thimble specifically designed for hosting the ATI-Nano17. This configuration enables thimble measurement calibration.

The thimble requires a new calibration in addition to the calibration undertaken for each sensor. This calibration aims to determine the precision with which the thimble applies normal and tangential forces. Thus, it is important to compare anew the data obtained from the thimble sensors with the data from F/T sensor of ATI Nano 17. This comparison allows us to determine the quality of the estimated normal and tangential forces. A new thimble containing both the ATI-Nano17 and the four contact force sensors was designed, as shown in Figure 7. This new thimble was specifically designed to compare information provided by the thimble sensors to the ATI-Nano17 [22]. Normal forces are provided by the sensor located at the fingertip of the thimble (sensor 1 in Figure 2), and correspond to the information provided by the ATI-Nano17 in the Z-Axis direction. The tangential forces are obtained from the data provided by three sensors in the thimble (sensors 2, 3, and 4 in Figure 2), and correspond to the data provided by the ATI-Nano17 in the X–Y plane.

The difference between the data provided by both sensors (thimble and ATI-Nano17) is approximately ±1.43 N with a range of 0 to 20 N. Depending on the application, this deviation may or may not be deemed acceptable. For instance, in the case of training tasks in rehabilitation applications, it is acceptable because the forces applied between different practitioners vary significantly and do not require a higher level of precision [23]. For applications in which this resolution is insufficient, an adapted thimble can be used to which a high precision force/torque sensor can be incorporated. It is important to remember that this will significantly increase the price and inertia of the device. The signal variation was also checked. This comparison concerns precision in the detection of force flanks. In this case, the first derivatives of data provided by both sensors are compared. These derivative functions are calculated as follows:

(2) $$f_{i}^{\prime }=\Delta f_{i}/\Delta t=\left(f_{i}-f_{i-1}\right)/\Delta t$$

**Figure 8.**
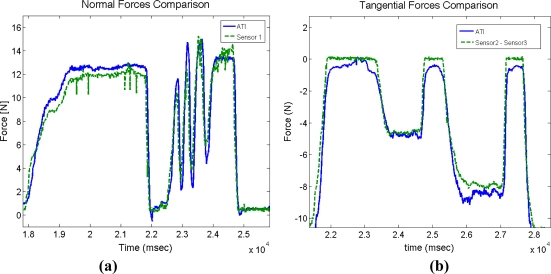
Thimble calibration. **(a)** Comparison between normal forces measured by ATI and Flexiforce sensors located at the finger base. **(b)** Comparison between tangential forces measured by ATI and Flexiforce sensors located at the finger sides.

The standard deviation of the difference between both derivatives is equal to ±0.166 N/s. Consequently, the estimation of manipulation force flanks is much more precise than the estimation of normal and tangential forces. As shown in Figure 8, it can be observed that when forces vary, the flanks of the signals measured by the thimble and the corresponding signals provided by the ATI-Nano17 are very similar. This information may be useful for some applications such as task segmentation, event or contact detection, *etc*.

## 5. Applications

The estimation of both normal and tangential forces while performing advanced manipulation has proven to be useful for several applications including segmentation of manipulation tasks and fast modeling of virtual objects.

- **Task segmentation:** Attention to the derivative of the force signal shows that the task can be segmented into different stages which may be useful for a range of applications including those that improve adaptive control architectures and those that bringing attention to an omitted step.
  - Control parameters can be optimized for different stages of the task at hand or for transmitting a unit of force whenever an event occurs, as opposed to transmitting the exact manipulation force which can be physically demanding for the operator in given situations.
  - Bringing attention to omitted steps in a previously defined task can increase safety in tele-maintenance operations or to assist people to perform a daily living task, as in the performance of common tasks.
- **Modeling physical interactions**: Information obtained in relation to the forces experienced by a user while manipulating a complex object can contribute to the design of a virtual model of this physical interaction. The physical modeling of the forces of a complex system in order to generate a virtual model with which the user can interact by means of a haptic device can result in complex equations that the system must solve in real time, hence requiring high performance computers and GPUs. In contrast, this proposed design shows that manipulation forces can be recorded and translated into a simulation model such as a look up table or a simple interpolation reduces the hardware requirements for haptic applications.

The following section shows various applications of the sensorized thimble for segmentation and modeling virtual environments.

### 5.1. Segmentation of Real Tasks: Manipulation of a Bottle Containing Liquid

**Figure 9.**
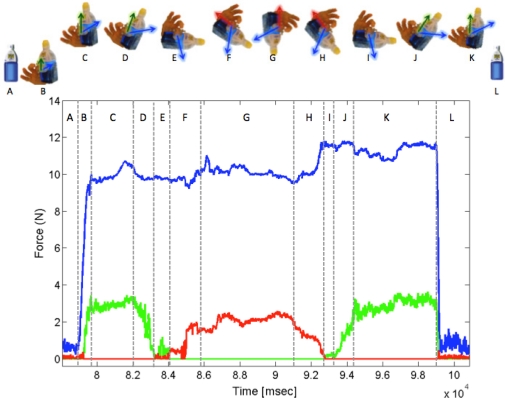
Task segmentation during the manipulation of a bottle containing liquid. Figures above show different segmented stages of the task and the figures below show the recorded forces. (A) Approaching the bottle. (B) Grabbing the bottle increasing the force. (C) Lifting the bottle vertically. (D) Tilting the bottle. (E) Holding the bottle in a horizontal state. (F) Tilting the bottle. (G) Holding the bottle upside down. (H) Tilting the bottle back. (I) Holding the bottle in a horizontal position. (J) Tilting the bottle back. (K) Holding the bottle in a vertical position. (L) Releasing the bottle.

Information provided by thimble sensors is more accurate for detecting flanks than absolute values. Therefore, certain experiments related to performance detecting flanks were undertaken. This subsection summarizes the results obtained in an experiment focusing on the manipulation of a recipient containing liquid. Given the available force information, the task can be segmented into different stages: approximation, contact, grasping, lifting, tilting, and releasing. An example of task segmentation is shown in Figure 9. This figure shows the different stages and the recorded forces when manipulating a bottle containing liquid. First, the user approaches the bottle (A). When the user is in the correct position, he or she grabs the bottle (B) by increasing the force until he or she is able to lift it (C). Then, the user holds the bottle vertically (D) and starts tilting it (E) until the bottle is in a horizontal state. The user continues by tilting the bottle and then tilting it back to the horizontal state (G). Finally, the user holds the bottle vertically again and releases it over the table (I). This information can also be used to create an approximate model of this system [22], and results in the reduction of complex model equations that the system must solve in real time and that require high performance computers and Graphic Processing Units (GPUs).

### 5.2. Modeling Physical Interactions: Body-Joint Model for Medical Rehabilitation Simulators

**Figure 10.**
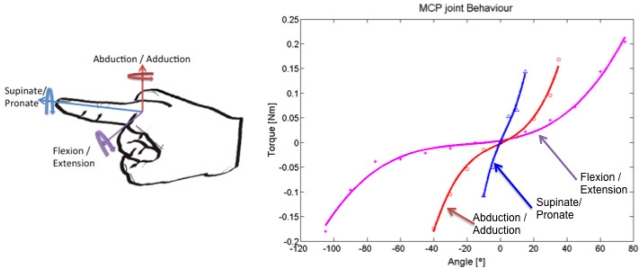
Data obtained for the metacarpal joint of the index finger. This data concerns the three possible rotations in the MCP joint.

The sensorized thimble that is described here can be used for creating an approximate model of the characteristics of human joints, which are inherently multidimensional and non-linear [23]. The system developed characterizes the stiffness of the metacarpo-phalangeal joint, which is located at the index finger in the axes of rotation. Note that even though the finger can mainly move in the flexion/extension and abduction/adduction angles of rotation, for rehabilitation of the finger, the pronate/supinate degrees of freedom (DoF) should also be considered. The finger was mobilized in the whole range of movements and the force was saved for different angles of the finger; a minimal square polynomial interpolation was calculated to approximate the joint’s behavior in every rotational DoF, as shown in Figure 9. Equations (3)–(5) show the relationship between the angles and torques (force was applied at approximately 1.2 cm from the center of rotation):

(3) $$\tau \left(\alpha \right)=2.2e-7\cdot \alpha^{3}+1.24e-05\cdot \alpha^{2}+5.56e-04\cdot \alpha +7.1e-3$$

(4) $$\tau \left(\beta \right)=2.04e-6\cdot \beta^{3}+1.44e-5\cdot \beta^{2}+1.6e-3\cdot \beta -5.9e-3$$

(5) $$\tau \left(\gamma \right)=1.68e-5\cdot \gamma^{3}-1.68e-4\cdot \gamma^{2}+7.8e-3\gamma +1.1e-3$$

where α represents the flexion/extension angle, β represents adduction/abduction angle, and γ represents the pronate/supinate angle, as shown in Figure 10.

The minimum square error for these polynomial interpolations is 5.8e-3 Nm, 4.6e-3 Nm, and 8.6e-4 Nm, respectively, which are less than the thimble’s error. These results are similar to those of previous found in the vitro study of flexion/extension stiffness using freshly frozen fingers from a cadaver [24]. This model was implemented in a simulator and potentially can be used by students to learn and practice rehabilitation procedures.

## 6. Conclusions

Researchers agree that force sensing at the end-effector of haptic devices improves the performance of real and virtual object manipulation. However, current commercially available impedance-type haptic devices do not include force sensing capabilities due to the high price and weight of traditional high precision sensors. This paper describes the design and calibration of a lightweight and cost-effective end-effector that can be adapted to anatomical variations among users and attached to commercially available haptic devices. Contact force sensors only measure forces applied in the normal direction to its active surface. As previously described, both normal and tangential contact forces can be estimated by strategically positioning four contact force sensors inside a thimble.

The proposed end-effector has a measurement error of ±1.43 N, which is sufficient for certain applications that capture and model virtual scenarios such as object grasping or rehabilitation applications. In these applications, forces applied between different practitioners vary significantly while still resulting in correct practices.

Also, we have shown that the proposed design is very precise in the performance of force signal edge detection (±0.166 N/s). Force edge information can be used for task segmentation, which is useful in the undertaking of a complex manipulation. This task segmentation allows us to determine which stage a user is at of an overall task. This segmentation can be useful for reminding the user if he or she skips a step of a task or for teleoperation by only transmitting to the user force information based on events.

Moreover, segmentation can also describe an application for real object manipulation. This application focuses on modeling the mechanical features of finger joints; in particular, the stiffness of the metacarpo-phalangeal finger joint in the three directions. It demonstrates that information provided by the sensorized thimble can also be applied towards manipulation of real objects. The development of these kinds of models is useful for reproducing this type of manipulation in a more realistic manner.
